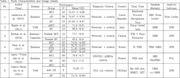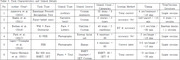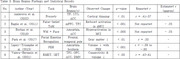# Neuroimaging‐Based Assessment of Social Cognition in Older Adults with Mild Cognitive Impairment: A Systematic Review

**DOI:** 10.1002/alz70861_108991

**Published:** 2025-12-23

**Authors:** Gyeomju Han

**Affiliations:** ^1^ Yonsei University, Wonju‐si, Gangwon‐do, 1, Yeonsedae‐gil, Heungeop‐myeon Korea, Republic of (South)

## Abstract

**Background:**

Social cognitive impairments, including Theory of Mind (ToM) deficits and emotion recognition deficits, may emerge during the early stages of mild cognitive impairment (MCI). Advances in neuroimaging enable the identification of neuroanatomical correlates associated with these impairments. This systematic review synthesized neuroimaging findings related to social cognition in individuals with MCI.

**Method:**

Following PRISMA guidelines, studies utilizing computer‐based ToM or emotion recognition tasks and MRI or fMRI assessments were included. Extracted data included task characteristics, imaging modalities, and associations between cognitive performance and brain structure.

**Result:**

Deficits in ToM were associated with reduced activation in the medial prefrontal cortex (mPFC) and temporoparietal junction (TPJ). Deficits in emotion recognition correlated with decreased gray matter volumes in the amygdala, fusiform gyrus, and pallidum (r = –.39 to –.50, *p* < .001), and findings remained significant after correction for multiple comparisons.

**Conclusion:**

Social cognitive impairments in MCI are linked to distinct neuroanatomical changes. Neuroimaging‐based assessments targeting social cognition may support the early identification of dementia risk and inform the development of intervention strategies